# Comparative evaluation between guided endodontics and conventional technique: A prospective clinical study

**DOI:** 10.6026/973206300222353

**Published:** 2026-04-30

**Authors:** Vaibhav Sharma, Pranjali Dutt, Endla Varun Kumar, Shalaka Raurale, Tanvi Saraf, Apoorva Sharma, Miral Mehta

**Affiliations:** 1 Department of Conservative Dentistry and Endodontics, Shri Balaji Institute of Dental Sciences, Raipur, Chhattisgarh, India; 2 Department of Dentistry, Mahamaya Rajkiya Allopathic Medical College, Ambedkarnagar, Uttar Pradesh, India; 3 Department of Dentistry, Maharaja's Institute of Medical Sciences, Nellimarla, Vizianagaram, India; 4 Department of Orthodontics and Dentofacial Orthopaedics, Dr. N. Y. Tasgaonkar institute of medical science and Research Center, Diksal, Karjat, India; 5 Department of Pediatric and Preventive Dentistry, Bharati Vidyapeeth (Deemed to be university) Dental College and Hospital, Navi Mumbai, India; 6 Department of Pediatric and Preventive Dentistry, Karnavati School of Dentistry, Karnavati University, Gandhinagar, Gujarat, India

**Keywords:** Guided endodontics, pulp canal obliteration, calcified canals, 3D-printed guide, conventional endodontics, minimally invasive

## Abstract

Calcified root canals challenge endodontic success through procedural errors, excessive dentin loss and treatment failure when managed conventionally. Therefore, it is of interest to compare guided endodontics versus freehand technique in 60 teeth (48 patients) with pulp canal obliteration (n=30/group). Guided endodontics achieved superior canal location (96.7% versus 73.3%), reduced negotiation time, minimized dentin removal and lowered procedural complications (perforation/ledge/instrument separation). Patient-reported outcomes favored guided endodontics at 1 week and 3 months, with less post-operative pain and higher satisfaction. This data helps to guide endodontics for precise, minimally invasive standard in treating calcified canals, thereby transforming the management of historically problematic cases.

## Background:

Pulp canal obliteration is one of the pathological conditions, which is manifested by deposition of mineralized tissue in the root canal system with the result of partial or total constriction of the pulp chamber and the root canal lumen [1]. It is an outcome of dental trauma, most often luxury, but also old age, chronic inflammatory stimulation, orthodontic therapy and some restorative treatment [2]. According to epidemiological evidence, pulp canal obliteration is estimated to be in the range of 4-24 percent of traumatized teeth and the rate is higher when the traumatic injuries are severe and of a type [3]. Treatment of teeth with obliterated canals has proved to be a formidable clinical challenge in endodontic management. The reduced or absent visualization of the canal space on radiographic images complicates treatment planning and dense, calcified tissue encountered during the creation of access cavities increases the risk of errors during the procedure, such as root perforation, over-removal of dentin, ledges and instrument separation [4]. Research studies have reported perforation rates of 4 to 10% with the traditional method of treating calcified teeth in endodontics and the overall treatment success has been very low compared to treatment of patent canals [5]. The traditional endodontic treatment of calcified canals is based on either the clinician's anatomic knowledge or the clinician's tactile sense and on the use of magnification devices, such as dental operating microscopes and ultrasonically powered tools, to find and navigate the obliterated canal [6]. Although the use of a dental operating microscope has greatly enhanced visualization during these processes, the process is highly operator-based. It has inherent spatial orientation constraints, especially when the calcification has penetrated deep into the root canal system [7]. Moreover, the large amount of exploratory dentin loss that may occur during conventional access preparation predisposes the tooth to fracture and may affect the long-term prognosis [8]. Guided endodontics was developed as a logical follow-up to the concept of computer-aided implant surgery in endodontics. The technique was first reported in 2014 as a new method for handling more complex endodontic situations, combining cone-beam computed tomography views with digital surface scanning to develop a virtual endodontic treatment plan, which is then converted into a three-dimensional printed drilling guide [9]. This guide will set the course of a specialized trephine bur along a strictly defined path from the tip of the tooth to the target canal, permitting the discerning and predictable positioning of canals and reducing unnecessary dentin loss [10]. Guided endodontic access consists of a series of digital operations: acquisition of a high-resolution scan of the CBCT, generation of a digital surface model with the use of intraoral scanning or scanning of a stone model, overlay of the two sets of data in special planning programs, planning of the optimal course of drilling virtually and printing a physical guide with stereolithographic three-dimensional printing [11]. The accuracy of this workflow has been demonstrated in several *in vitro* studies, showing angular deviations of less than 2 degrees and linear deviations at the apex of less than 1 millimeter [12]. Although guided endodontics has yet to be demonstrated to be effective in clinical applications, recent clinical experiences and case series have shown promising results with guided endodontics in more challenging cases, such as teeth with calcified canals, teeth requiring endodontic retreatment with a prosthetic restoration and apical surgery with meticulous osteotomy planning [13]. Nevertheless, the available literature mostly focuses on laboratory research using extracted teeth and three-dimensional-printed prototypes and relatively little prospective clinical research has compared the results of guided endodontics with those of traditional freehand methodology [14]. Further, the majority of published clinical data are single case reports or small case series, which lack the statistical power to draw strong conclusions about the comparative benefits of guided endodontics [15]. There is a critical gap in research on the systematic clinical comparison of guided endodontics and conventional methodology, using a variety of outcome measures, not just technical accuracy, but also procedural efficiency, complication rates, dentin conservation and patient outcomes [16]. Therefore, it is of interest to compare and contrast the clinical results of guided endodontics versus the traditional freehand technique in the endodontic treatment of pulp-canal obliterated teeth, including canal location success rate, procedure time, dentin removal volume, rate of procedural errors, precision of trajectory and patient outcomes.

## Materials and Methods:

## Design of the study and ethical approval:

This was a prospective, parallel-group, randomized comparative clinical trial conducted in the Department of Conservative Dentistry and Endodontics over 20 months.

## Sample size calculation:

The pilot data comprised eight cases, for which the sample size was calculated using G*Power (version 3.1.9.7). The guided group was assumed to have a canal location success rate of 95% and the conventional group of 70%; an alpha error of 0.05 and a statistical power of 80 would require a minimum of 26 teeth in each group. Thirty teeth were assigned to each group to balance potential dropouts and protocol breaches, yielding a total of 60 teeth.

## Participant selection:

The outpatient clinic recruited forty-eight patients with sixty teeth who had pulp canal obliteration. Pulp canal obliteration was diagnosed through clinical examination that showed yellow discolouration of the crown and periapical radiographs showing partial or complete canal obliteration, confirmed by CBCT examination.

## Inclusion criteria:

Permanent anterior or premolar teeth that had pulp canal obliteration (either Type II or Type III as per the American Association of Endodontists classification), the need to perform endodontic therapy because of periapical pathology or elective therapy before undertaking the prosthetic rehabilitation, age (18 to 65 years) and willingness to undergo the scheduled follow-up appointment.

## Exclusion criteria:

Teeth with root resorption larger than one-third of root length, teeth with open apices, teeth with vertical root fractures, teeth with severe periodontal involvement (probing depth larger than 6 mm), pregnant or lactating patients and teeth where the residual canal lumen was completely obliterated in all axial slices in all cases with CBCT evaluation.

## Randomization and group allocation:

Randomization was performed by assigning eligible teeth to one of two groups (randomization sequence generated by computer), with block randomization (block size was four) and concealed allocation (from sequentially numbered, sealed, opaque envelopes).

[1] Group I - Guided endodontics (n = 30): Group I Participants are treated with Access cavity preparation in a 3D-printed surgical guide.

[2] Group II - Conventional technique (n = 30): Freehand preparation of access cavity under magnification under the dental operating microscope.

## Group I - Pre-operative assessment and digital planning:

Both groups of patients were provided with a preoperative CBCT scan using a standardized acquisition setting (field of view: 5 x 5 cm, voxel size: 0.125 mm, tube voltage: 90 kVp, tube current: 8 mA, exposure time: 12 seconds). In Group I patients, an intraoral scanner (TRIOS 3, 3Shape, Copenhagen, Denmark) was also used to obtain a high-resolution surface representation of the dental arch as a digital impression. The intraoral scan file (STL format) and CBCT dataset (DICOM format) were then imported into special implant/endodontic planning software (coDiagnostiX, Dental Wings, Montreal, Canada). The two datasets were overlaid using a surface-matching algorithm and then refined by hand. The best path for drilling was determined by identifying the maximum coronal visible remnant of the canal on axial CBCT slices and establishing a linear course between the proposed access point on the tooth surface and the target point, avoiding the root surface by a minimum line of 1 mm. The drilling depth was calculated and a virtual sleeve on the guide was created and placed. The final guide design was exported as an STL file and printed in three dimensions (3D) using a stereolithographic 3D printer (Form 3B, Formlabs, Somerville, MA, USA) with a biocompatible dental resin (Surgical Guide Resin V1, Formlabs). The post-processing steps entailed 15 minutes in 99% isopropyl alcohol and 30 minutes in a UV light chamber at 60°C. The internal diameter of the trephine bur chosen was bonded to the metal guide sleeves using cyanoacrylate adhesive.

## Clinical procedures:

[1] Group I (Guided Endodontics): The 3D-printed guide was placed over the dental arch after administration of a local anesthetic and placement of a rubber dam. Profitability and viability were established before the drilling process commenced. Access was obtained using a special trephine bur (Munce Discovery Burs, CJM Engineering, Santa Barbara, CA, USA) at a constant speed of 800 rpm, with sterile saline irrigation maintained throughout. The bur was then positioned inside the guide sleeve to the set depth. Once the target had been met, the guide was removed and the canal was opened with a size 08 or 10 K-file.

[2] Group II (Conventional Technique): The access cavity preparation was performed with round diamond burs in a high-speed handpiece under a dental operating microscope (OPMI PROergo, Carl Zeiss, Oberkochen, Germany) at 1016x magnification immediately after anesthesia and rubber dam isolation. Primary penetration was performed with a round bur and ultrasonic tips (Start-X, Dentsply Sirona, Ballaigues, Switzerland), which were then explored at 35 W under constant irrigation. The location of the canals was attempted using small K-files (sizes 06, 08 and 10) with frequent irrigation and tactile examination. The operating procedure was terminated and declared unsuccessful after 45 minutes if the canal was not found or if a hole was found.

## Post-operative management:

When the root canal location was successful, it was negotiated, debrided with a rotary nickel-titanium file system (ProTaper Gold, Dentsply Sirona), shaped and obturated with warm vertical condensation. Standardized postoperative instructions were provided to all patients and analgesia was prescribed on demand.

## Outcome measures:

[1] Canal location success rate: Diagnostic finding and negotiation of the root canal to within 1 mm of the working length and undetected perforation.

[2] Total procedural time: The duration of time taken to make the access preparation to the confirmed canal patency in minutes with a digital stopwatch.

[3] Volume of dentin lost: Evaluated by summing up the pre-operative and postoperative volumes of CBCT scans and finding the volume of access cavity with the aid of image analysis programs (Mimics, Materialise, Leuven, Belgium).

[4] Procedural error incidence: Perforation, ledge formation and separation of instruments, both clinically assessed and confirmed radiographically.

[5] Off-course (Group I only): Determined by the comparison between the planned virtual path and the real bur path on the post-operative CBCT, angular deviation (degrees) and linear deviation of the tip (mm).

[6] Patient-reported outcomes: Intensity of post-operative pain measured by the 100-mm visual analog scale at 24, 48 and one-week intervals and overall patient satisfaction measured on a five-point Likert scale at 3 months.

## Statistical analysis:

The SPSS software (version 27.0, IBM Corporation) was used to perform the statistical analysis. Normality of data distribution was tested using the Shapiro-Wilk test. An independent samples t-test was used to compare normally distributed continuous variables, whereas the Mann-Whitney U test was used to compare non-normally distributed continuous variables. The chi-square test or Fisher's exact test was used to analyze categorical variables. The statistical significance was considered at p < 0.05.

## Results:

All sixty teeth in forty-eight patients completed the treatment phase. No patients withdrew from the study during the treatment period and all participants attended the scheduled follow-up appointments. The demographic characteristics were comparable between groups, with no significant differences in mean age (Group I: 34.8 ± 11.2 years; Group II: 36.4 ± 10.7 years; p = 0.574), sex distribution (p = 0.712), or tooth type distribution (p = 0.648). The guided endodontics group achieved a significantly higher canal location success rate of 96.7% (29 of 30 teeth) compared to 73.3% (22 of 30 teeth) in the conventional technique group (p = 0.023). The single failure in the guided group was attributed to guide instability during drilling of a mandibular premolar with a short clinical crown. The eight failures in the conventional group included four cases where the canal could not be located within the allotted 45 minutes and four cases complicated by procedural perforation. The mean procedural time from initiation of access preparation to confirmed canal patency was significantly shorter in the guided group compared to the conventional group (p < 0.001). Furthermore, the volumetric analysis of dentin removed during the access procedure demonstrated significantly less tissue sacrifice in the guided endodontics group (p < 0.001) (Table 1 - see PDF). The incidence of procedural errors was significantly lower in the guided endodontics group compared to the conventional technique group. No perforations occurred in the guided group, while four perforations (13.3%) were recorded in the conventional group. Ledge formation occurred in 1 case (3.3%) in the guided group, compared with 5 cases (16.7%) in the conventional group. No instrument separations occurred in either group. The overall complication rate was significantly different between groups (p = 0.038). For the guided endodontics group, trajectory accuracy analysis revealed high precision in both angular and linear deviation measurements. The mean angular deviation between the planned and actual drilling trajectory was 1.38 ± 0.72 degrees. The mean linear deviation at the coronal entry point was 0.29 ± 0.14 mm, while the mean linear deviation at the apical target point was 0.47 ± 0.21 mm (Table 2 - see PDF). Post-operative pain levels were comparable between the two groups at 24 hours and 48 hours, with no statistically significant differences. However, by the one-week follow-up, Group I demonstrated significantly lower mean VAS pain scores compared to Group II (p = 0.031). At the three-month evaluation, patient satisfaction was significantly higher in the guided endodontics group, with 86.7% reporting being "very satisfied" or "satisfied" compared to 63.3% in the conventional group (p = 0.029) (Table 3 - see PDF).

## Discussion:

This prospective comparative study provides solid clinical evidence that guided endodontics is superior to conventional freehand methods in the treatment of teeth with pulp canal obliteration. The much higher rate of successful canal placement, the shorter procedure times, the lower dentin loss and the lower complication rate observed in the guided group all demonstrate the clinical benefits of integrating digital planning and guided access in endodontic practice to tackle complex cases. The 96.7% success rate for guided endodontic canal location in the current study is comparable to the 92-100% success rates reported in recent systematic reviews and clinical trials, which have reported successful cases of guided endodontics across a variety of clinical situations necessitating canal location [17]. Such a high success rate is indicative of the inherent benefit of the guided method, namely its ability to accurately target residual canal remnants that may not always be visible under direct visualization but can be identified on high-resolution CBCT imaging [18]. Conversely, the conventional group success rate of 73.3%, although similar to earlier reported scores for freehand navigation of severely calcified canals, underscores the inherent constraints of these tactics, which rely on haptic cues and visible marks to navigate dense mineralized tissue [19]. The large difference in procedure times between the two groups warrants careful interpretation. Although the guided group required a much shorter clinical chairside time for the access and canal location procedure, it failed to reflect the time spent on digital planning, guide design and fabrication. Past studies of the entire guided endodontic process have estimated that the digital planning phase adds 30-60 minutes of lab time [20]. The resulting pre-operative investment, however, yields less time for clinical procedures, more predictable results and possibly a reduction in operator stress during the actual treatment, especially in more complicated canal-searching cases that take over an hour [21]. One of the most important clinical implications of this study is the volumetric analysis of dentin removal. The group-guided also showed over 56 per cent less dentin loss than the conventional group and this has far-reaching implications on long-term tooth survival. It is widely reported that over-preparation of endodontic access removes a large amount of dentin, significantly reducing the fracture resistance of endodontically treated teeth [22]. The idea of minimally invasive endodontics, which postulates the maintenance of pericervical dentin as a key factor influencing the structural integrity of the tooth, is naturally lent by the guided approach, which establishes a narrow and specifically directed access opening instead of the wide exploratory cavity usually needed in the traditional treatment of the calcified canals [23]. The number of perforations in the guided group is zero, whereas the conventional group has four, indicating the safety benefits of guided access preparation. Root perforation during canal searching is also a major complication, significantly worsening the prognosis of the impacted tooth and potentially requiring further surgery or extraction [24]. The observed perforation rate of 13.3% in the conventional group, although worrisome, is comparable to that reported in the published literature for freehand operation of totally calcified canals [25]. This virtual pre-operative planning inherent in guided endodontics, which involves confirming the drilling path relative to the root boundaries with a specified safety margin, is an effective way to mitigate the risk of accidental perforation, provided the guide is properly fabricated and well-positioned during the procedure [26]. The results of the trajectory accuracy measurements in the guided group showed angular and apical linear deviations equal to those measured in validated laboratory tests, which proves that the precision attained in the controlled laboratory environment could be reliably applied to clinical practice [27]. The angulation deviation of 1.38 degrees and apical linear deviation of 0.47 mm in this study are within the clinically acceptable range, bearing in mind that the majority of the root canals in the target level are more than one millimeter in diameter [28]. There is a note that minor deviations can become clinically significant in teeth with incredibly thin roots or complex anatomical structures, because even a sub-millimeter error might lead to perforation or canal transportation [29]. The interesting pattern in the patient-reported outcomes was that the patient had the same pain levels in the early post-operative period, but much lower pain in the guided group at 1 week. Probably, this difference at later stages is related to lower tissue damage and inflammatory reaction with the more conservative guided access, because less dramatic dentin removal and reduced time spent during the procedure reduce mechanical and thermal injury to the periradicular tissues [30]. The high patient satisfaction rate in the guided group may be explained by the combination of successful cure rates, low complication rates and the assumption that patients received technologically advanced care [31]. This study has several shortcomings that warrant mention. This study was not blinded because the two methods differed in nature, which could have introduced assessment bias, especially for subjective outcome measures. Three months of follow-up is insufficient to assess the long-term success of endodontic treatment, as at least 1 year of radiographic follow-up is needed to evaluate periapical healing [32]. Also, the research did not include teeth that were completely obliterated by canals in all axial CBCT cuts; the study sample did not represent the extreme cases of calcification. Guided endodontics cost-effectiveness was not evaluated formally and the extra costs of acquiring CBCT, intraoral scanning, licensing software and 3D printing will have to be contrasted with the clinical utility when making a treatment recommendation [33]. New opportunities are emerging in the field of guided endodontics, such as the development of dynamic guidance systems that do not require a physical drilling template to provide real-time intraoperative guidance and the optimization of artificial intelligence algorithms to plan trajectories [34] automatically. With these technologies becoming increasingly accessible, promising signs of guided endodontic methods are expected to extend beyond the effect of calcified canals to include a larger number of intricate endodontic cases.

## Conclusion:

Guided endodontics achieved superior canal location success (96.7% versus 73.3%), reduced procedure time, minimized dentin removal and eliminated procedural complications compared to conventional freehand access. Patient-reported outcomes favored guided endodontics with less postoperative pain at 1 week and higher satisfaction at 3 months, confirming its clinical acceptability. Thus, guided endodontics is the precise, minimally invasive standard for pulp canal obliteration, warranting integration into clinical practice pending long-term cost-effectiveness validation.
